# Publisher Correction: Abstracts from the 7th European Society of Tissue Regeneration in Orthopaedics and Traumatology (ESTROT)

**DOI:** 10.1007/s00068-024-02614-8

**Published:** 2024-09-05

**Publisher Correction: European Journal of Trauma and Emergency Surgery**

10.1007/s00068-024-02489-9.

The article “Abstracts from the 7th European Society of Tissue Regeneration in Orthopaedics and Traumatology (ESTROT)” was published on April 25, 2024 without Open Access on the publisher’s internet portal due to an error by the publisher. The copyright of the article has changed to © The Author(s), under exclusive licence to Springer-Verlag GmbH Germany 2024 and the article is forthwith distributed under a Creative Commons Attribution 4.0 International License, which permits use, sharing, adaptation, distribution and reproduction in any medium or format, as long as you give appropriate credit to the original author(s) and the source, provide a link to the Creative Commons licence, and indicate if changes were made. The images or other third party material in this article are included in the article’s Creative Commons licence, unless indicated otherwise in a credit line to the material. If material is not included in the article’s Creative Commons licence and your intended use is not permitted by statutory regulation or exceeds the permitted use, you will need to obtain permission directly from the copyright holder. To view a copy of this licence, visit http://creativecommons.org/licenses/by/4.0/.

Furthermore, the title page of the abstract was only included in the PDF version of the article.

The publisher apologizes for the error.

The original article has been corrected.

